# Exploring Shyness among Veterinary Medical Students: Implications for Mental and Social Wellness

**DOI:** 10.3390/vetsci5020056

**Published:** 2018-06-15

**Authors:** Kenneth Royal, Mari-Wells Hedgpeth, Keven Flammer

**Affiliations:** 1 Department of Clinical Sciences, North Carolina State University, Raleigh, NC 27607, USA; 2 Office of Academic Affairs, North Carolina State University, Raleigh, NC 27607, USA

**Keywords:** shyness, wellness, student health, higher education, veterinary medicine, medical education, psychology, social phobia, personality, mental health

## Abstract

Background: Shyness is defined as “the tendency to feel awkward, worried or tense during social encounters, especially with unfamiliar people.” While shyness is not necessarily a social disorder, extreme cases of shyness may classify as a social phobia and require medical treatment. Extant research has noted shyness may be correlated with social problems that could be detrimental to one’s health, career, and social relationships. This exploratory study examined the prevalence, source, and nature of shyness among incoming Doctor of Veterinary Medicine (DVM) program students at one veterinary medical school. Methods: One hundred first-year DVM program students were administered a modified version of the Survey on Shyness. Results: Results indicate most students (85%) self-identified as at least a little shy, a figure that is believed to be significantly higher than national population norms in the United States. Students attributed the primary source of shyness to personal fears and insecurities. Students reported frequent feelings of shyness and generally perceived shyness as an undesirable quality. Students reported that strangers, acquaintances, authority figures, and classmates often make them feel shy. Conclusions: Given the high prevalence of self-reported shyness among veterinary medical students, institutions may wish to include strategies to address shyness as part of a comprehensive wellness program.

## 1. Introduction

The American Psychological Association (APA) defines shyness as “the tendency to feel awkward, worried or tense during social encounters, especially with unfamiliar people” [1]. In 1975, 40% of Americans were estimated to be ‘shy’, a figure that subsequently rose to 48% in 1995 [2,3]. While shyness is not necessarily a social disorder, extreme cases of shyness may classify as a social phobia and require medical treatment. Research suggests, however, that only a small fraction of shyness cases qualify as a social phobia [4].

Shyness often is confused with introversion, as both appear quite similar on the surface. The primary difference between a shy person and an introvert is an introvert prefers solitude, whereas a shy person wants to be sociable but is too uncomfortable to achieve it [5]. Although shy people typically are introverts, extroverts may also be shy. These individuals, often politicians, celebrities, and college professors (among others), have high self-esteem and good social skills but often are uncomfortable interacting with strangers. According to Carducci and Zimbarro [2], only 15–20% of shy people fit the stereotypical shy personality. Pilkonis [6] referred to the remaining 80–85% as ‘privately shy’ people. Privately shy people exhibit good social skills and seem confident, but internally are experiencing negative thoughts about themselves. Others report feeling shy only in certain situations, thus environmental and situational factors also tend to play a role in how often individuals experience feelings of shyness. Therefore, it is important to note that shyness is quite complex and the extent to which an individual is shy may be highly variable, and context-specific, across a continuum of shyness. 

In recent years, the ‘medicalization’ of shyness has received a great deal of attention. Shyness shares characteristics but is distinct from social anxiety disorder (social phobia), a recognized medical problem that is listed in the Diagnostic and Statistical Manual of Mental Disorders [7]. While shyness is common, the 12-month prevalence for social anxiety disorder in the US is only 7% of the US population and only 12% of self-identified shy people meet the diagnostic criteria for social anxiety disorder [8]. Persons with social anxiety disorder experience much greater impairment and often require medical and/or psychological treatment. Dalrymple and Zimmerman [9] stress that it is important to distinguish normative shyness from social anxiety disorder to avoid stigmatization and unnecessary treatment of shy individuals. 

Research on the biomedical factors related to shyness is quite diverse. For example, neurophysiological studies have found discernible differences in brain behaviors for shy adults. More specifically, shy adults tend to exhibit greater relative right frontal electroencephalogram (EEG) activity [10] and have higher levels of activity in the amygdala [11]. Similarly, studies have also shown that people with high levels of shyness tend to have lower baseline cortisol levels, which is thought to be a byproduct of a chronic increase in cortisol as a stress response to shyness during childhood [12,13,14]. Studies have also found that infants who exhibited social distress when surrounded by unfamiliar people and objects also exhibited symptoms of shyness years later [15]. Some studies have also suggested that shyness may be genetic [16,17,18]. 

Other scholars, however, have found that shyness largely is learned and is changeable. Some medical sociologists have described shyness as a symptom of cultural anxiety that results from modern societal expectations [19,20]. Scott S. [20] (p. 149) says “the stereotypical ‘symptoms’ of quietness, timidity and social withdrawal pose a significant challenge to the values of assertiveness, emotional literacy and vocal self-expression that pervade contemporary Western culture.” Scott goes on to say, “as shyness becomes less and less socially acceptable, the ‘shyest’ people are finding that their erstwhile deviant identities are being recast in biomedical terms and subjected to psychiatric treatment” (e.g., cognitive-behavioral therapy (CBT), self-help books, etc.), as well as pharmacological treatments (e.g., MAO-Is, beta blockers, benzodiazepines, and selective serotonins reuptake inhibitors (SSRIs)). Feinmann J. [21] (p. 47) argued that shyness may be reaching “epidemic proportions as quiet, introspective types increasingly see themselves as having a problem in a competitive, pushy culture”. Studies demonstrating the learned basis of shyness have suggested that controlling and overprotective parenting styles that involve frequent corrections and shaming tend to contribute the most [22]. Other studies have suggested that negative social experiences (e.g., embarrassment, failed relationships, emotional disappointments, etc.), traumatic events (e.g., divorce, getting fired from a job, etc.) and being labeled as shy (among other factors) also tend to play a role [2,23]. 

The negative impact of shyness can be quite costly. Excessive shyness has been linked to a number of negative outcomes, such as fewer interactions with others [24]; slower development of peer networks when entering college [25]; increased self-consciousness; [26] low self-esteem [27]; difficulty establishing new relationships [25]; and problems maintaining close relationships [24,25]. A study by Schmidt L.A. and Fox N.A. [28] found extreme shyness to be a strong predictor of a variety of mental health issues, such as depression, fearfulness, social anxiety, low self-esteem, loneliness, and psychosomatic issues such as gastrointestinal problems and allergies.

Research in evolutionary biology, however, suggests there are some advantages to being shy. Some scholars argue that shyness may come from prehistoric behaviors that helped aid survival. Animal studies involving the shy–bold continuum have found it often is advantageous to be timid and anxious. More specifically, while the more bold animals may acquire more mates and eat more food, shy animals that hide and avoid being social are less likely to be attacked, thus extending the length of their lives [29]. 

Extant research has noted a significant proportion of veterinary medical students tend to possess introverted personality types. More specifically, a study by Brown C.C. and colleagues [30] found 53% of veterinary medical students to be introverts, a number they say contrasts sharply with population norms of 35%. Given the strong relationship between introversion and shyness, as well as the prevalence for shyness in the general population, we sought to explore shyness in relation to veterinary medical students; a population that may be particularly vulnerable to feelings of shyness. At present, we are unaware of any research that has investigated shyness among any veterinary or medical student population. Therefore, this study sought to explore shyness and its relation to veterinary medical students.

## 2. Materials and Methods

### 2.1. Participants and Procedures

Each year at North Carolina State University College of Veterinary Medicine, all incoming Doctor of Veterinary Medicine (DVM) program students are administered an electronic survey during orientation. The purpose of the survey is to serve as a baseline measurement for a variety of measures and characteristics. Questions relate to students’ previous experiences, current attitudes and perspectives about the veterinary profession, future professional goals, and so on. The survey is part of a larger longitudinal assessment effort to measure student attributes over time. All data collected from the survey are treated with strict confidentiality. While the survey is voluntary in nature, repeated reminders by the college’s assessment team typically results in an excellent response rate. For the present study, all 100 incoming students completed the survey, resulting in a 100% response rate. The study was approved by the university’s Institutional Review Board (IRB).

Ages for incoming students ranged between 20 and 42, with a median age of 23 and a mean age of 24.64 (SD = 4.24). With respect to gender, 85 (85%) identified as female and 15 (15%) identified as male. With respect to race/ethnicity variables, 73 (73%) identified as White, 26 (26%) students identified as a member of one of 9 minority groups, and 1 (1%) student refused to identify this characteristic.

### 2.2. Instrumentation

A modified version of the Survey on Shyness [31], an instrument developed by the Shyness Research Institute, was utilized in this study. The survey began by providing the definition of shyness according to the American Psychological Association (noted previously) and followed with nine items, seven of which were categorical variables and two that were open-ended. Questions asked students to estimate the degree to which they self-identify as shy, how often they experience feelings of shyness, the impact (if any) of shyness with respect to their educational pursuits and personal lives, general perceptions of shyness, perceived sources of shyness, perceived ability to overcome shyness, and means to support veterinary students who are shy.

### 2.3. Analysis

Quantitative data analysis consisted of calculating descriptive statistics (e.g., frequencies and percents) via Statistical Package for Social Sciences (SPSS) software (version 24.0) (Ammonk, NY, USA) at both the overall and subgroup levels. Chi-squared tests were performed to determine if results differed by demographic subgroup. All statistical tests were performed with alpha set to 0.05. Qualitative data were initially analyzed via a content review by one author. Next, themes were extracted based on the classification of content. Codes were then generated for each discernible theme. A second author then evaluated qualitative content independently using the same coding schema. Both evaluators met to discuss any instances in which there was a discrepant result and revised ratings accordingly until a complete consensus was reached. 

## 3. Results

All quantitative results are presented in Table 1, Table 2 and Table 3. More specifically, Table 1 summarizes the results for items relating to general shyness. Table 2 summarizes results for the item inquiring if shyness has affected students’ educational experiences. Table 3 summarizes results for the item inquiring who makes students feel shy.

The students who indicated they were shy to some degree (n = 86) were presented an open-ended item asking about the source of their shyness. A total of 75 students provided comments with one predominant theme emerging. First, most students (n = 43, 57.33%) noted they have fears (e.g., rejection, being wrong, offending someone, being misunderstood, or disliked) and/or insecurities (e.g., lack of confidence/self-esteem). Other themes included personality/personal preference (n = 13, 17.33%), upbringing (n = 5, 6.67%), and genetic (n = 3, 4.00%). Nine (12.00%) participants were unsure about the source of their shyness, and 2 (2.67%) persons provided other answers. 

When asked what our College of Veterinary Medicine (CVM) can do to help persons who experience feelings of shyness, 75 (75%) students provided comments. The first theme involved students (n = 32, 42.67%) stressing the importance of a non-threatening, non-judgmental environment that is inclusive of everyone. The second theme involved students (n = 22, 29.33%) suggesting forced social opportunities (e.g., small group learning opportunities, required public speaking, etc.) would help students better cope with shyness. A smaller contingent of students (n = 9, 12.00%) noted the importance of getting to interact with faculty on an individual basis and non-forced social opportunities (e.g., student luncheons, etc.) (n = 3, 4.00%) would be helpful. Five (6.67%) students noted nothing could be done, and 4 (5.3%) were unsure.

All results were compared based on gender and race/ethnicity. Only one item revealed a statistically significant finding. Specifically, female students were more likely to self-identify as shy, χ^2^ (3) = 8.067, *p* = 0.045. This finding, however, should be interpreted with caution, as the subgroup size for male students was n = 15.

## 4. Discussion

### 4.1. Substantive Findings

Research has estimated that approximately 40–50% of Americans experience shyness [1,2]. Results from this exploratory study indicate most incoming veterinary students self-identify as being at least a little shy (84%), and only 16% of students stated they were not at all shy. On the surface, veterinary students appear to experience shyness at a much higher rate. However, it should be noted that the criteria for determining shyness between this sample and the United States population may be different, thus additional research is necessary to develop true comparisons.

Of those students that identified as shy, 27% reported experiencing feelings of shyness almost every day. Furthermore, 55% of students indicated shyness was at least an occasional personal problem for them. In general, most students (81%) view shyness as an undesirable quality, but most (86%) believe it can be overcome. When asked who makes students feel shy, strangers (73%), acquaintances (72%) authority figures (e.g., professors) (63%), and classmates (50%) were the most common responses, with family (6%) and close friends (0%) the least common. Students generally attributed their shyness to personal fears and insecurities. 

Pilkonis [6] noted 80–85% of shy people are “privately shy”. That is, they exhibit good social skills and appear confident, but internally are experiencing negative thoughts about themselves. Given most students noted their source of shyness was due to personal fears and insecurities, it is likely that most veterinary students also are “privately shy”. Also, as noted previously, extant research has identified linkages between extreme shyness and mental health issues (e.g., depression, fearfulness, social anxiety, etc.). It should be noted, however, that it is somewhat unclear exactly where the line between extreme shyness and social anxiety disorders end and begin. While the veterinary education community has devoted considerable attention to mental health and wellness issues in recent years, it also is advisable for veterinary educators to be cognizant of students who exhibit characteristics of excessive shyness as part of these efforts.

As noted previously, shyness may be correlated with social problems. In the present study, more than half of the students indicated shyness has affected their asking questions during class (55%), meeting new people (53%), and speaking up in class (52%). Thus, we argue that although veterinary programs tend to devote a great deal of attention to the intellectual and professional development of students, greater emphasis also should be placed upon social outcomes. This recommendation both is in alignment with students’ suggestions for supporting shy students and with extant research in higher education indicating additional and more intimate opportunities for students to engage with other students and faculty are more likely to improve a campus community [32]. Psychologists who help clients cope with shyness tend to promote the practice of “social fitness” as a key strategy. In much the same way that one must increase one’s activity level in order to improve their physical fitness, one must increase one’s social activity levels in order to improve their social fitness [33]. Social exercises consist of activities such as joining groups and communities, making new friends, and maintaining relationships with existing friends [33]. Interestingly, research on young adult populations has found the most common technique used by shy individuals to combat shyness is “forced extraversion”, which includes activities such as going to public places, joining clubs/groups, etc. [34]. Given many clinical psychologists encourage the practice of social fitness and many young adults already engage in activities to combat shyness, it would seem institutions that also focus on the social development of students may have an opportunity to help students potentially stave off a variety of negative outcomes (e.g., mental health, social relationships). 

One particularly interesting aspect of this study was that 16% of students noted that they were not at all shy. If one perceives shyness as a negative trait then one might contend that only 16% of students surveyed did not possess this undesirable quality. However, research has made it abundantly clear that shyness is not necessarily a bad thing. In fact, some evolutionary biologists have suggested there may be benefits to being shy. Therefore, one particularly interesting consideration, is that those students who report experiencing no shyness at all might actually be those individuals who are at the greatest disadvantage (e.g., more likely to exhibit narcissism, a lack of social awareness, over-confidence, etc.).

It is particularly important to note that the construct of shyness, like most latent traits, is unstable and subject to change. We contend that shyness should be thought of as a continuum onto which individuals can move back and forth based on various personal, environmental, and situational factors. Thus, it is possible for veterinary educators to help students better cope with feelings of shyness in the setting of their veterinary medical education. Some ideas to consider might include more simulated patient encounters with trained actors that can provide feedback, games during orientation to help facilitate relationships with students and faculty, frequent social events that convene students from multiple cohorts, friendly competitions (e.g., volunteer opportunities, charity events, athletic competitions), and so on.

One practical problem for addressing shyness involves how to bring up the topic with students. Research on shyness has indicated that some individuals may become shy as a result of being labeled as shy [1,23]. Our data indicate that most students feel being shy is an undesirable quality. Therefore, to mitigate this threat careful attention should be devoted to how to appropriately broach the subject with students. Thus, it would seem that any effort to combat shyness should be performed at the global level if shyness is acknowledged directly (e.g., a seminar on shyness and coping strategies). On the other hand, it likely would be appropriate to confront shyness at the individual level if students were approached more subtly (e.g., a social growth opportunity in which students are unaware that one of the intended outcomes is to increase their comfortability with others).

### 4.2. Future Research

This exploratory study opens the possibility for numerous lines of potential research relating to students and shyness. Some particularly worthwhile questions to consider include: To what extent does shyness impact students’ relationships with other students and with faculty? What educational (e.g., participation, etc.) and professional (e.g., contract negotiations, etc.) opportunity costs may be associated with shyness? What can institutions do to help shy students become more comfortable engaging in social activities? What measurements can best capture ‘private shyness’, perhaps the most common type of shyness?

Also, given veterinary faculty serve as the primary mentors and role models for students, it also would be worthwhile to explore shyness among veterinary faculty and the potential impact shy faculty may have on an educational environment. For example, do shy faculty typically have fewer interactions with students? Are shy faculty less involved as mentors and advisors? Do shy faculty utilize more passive instructional strategies? Do shy faculty offer fewer remediation opportunities? Are shy faculty more likely to acquiesce to student demands? Are shy faculty more likely to grade more leniently because they lack confidence in giving constructive feedback or want to avoid situations in which they must defend lower scores/ratings?

### 4.3. Limitations

This study possessed several limitations. Firstly, the study involves a single sample of incoming veterinary students at one institution. Also, all responses were self-reported by students. Finally, given this study is the first known study of its kind involving students in a health professions program there is a lack of literature to compare and contrast findings to discern where findings converge and diverge. It should be noted, however, that all incoming students participated in this study, thus non-response bias does not pose a validity threat to the findings. Furthermore, the confidential nature of the study and a promise of reporting data in aggregate form likely encouraged students to answer each question honestly, thus mitigating potentially dishonest or socially desirable answers.

## 5. Conclusions

In summary, this exploratory study examined the prevalence, source, and nature of shyness among incoming students admitted into a DVM program. Results indicate most students self-identify as at least a little shy with the primary source of shyness attributed to personal fears and insecurities. Students reported frequent feelings of shyness and generally perceived shyness as an undesirable quality. Students reported strangers, acquaintances, authority figures, and classmates often make them feel shy. These findings indicate that veterinary medical schools may wish to include strategies to address shyness as part of a comprehensive wellness program.

## Figures and Tables

**Table 1:** Frequency of responses to shyness items.

Item	Frequency
To what extent (if any) do you consider yourself to be a shy person?	
I am extremely shy	3 (3.0)
I am quite shy	21 (21.0)
I am a little shy	60 (60.0)
I am not at all shy	16 (16.0)
How often do you experience (have experienced) feelings of shyness?	
Every day	4 (4.0)
Almost every day	23 (23.0)
Often, nearly every other day	14 (14.0)
Once or twice a week	21 (21.0)
Occasionally, less than once a week	17 (17.0)
Rarely, once a month or less	21 (21.0)
How desirable is it for you to be shy?	
Very undesirable	42 (42.0)
Undesirable	39 (39.0)
Neutral	18 (18.0)
Desirable	1 (1.0)
Very desirable	0 (0.0)
How often would you say shyness had been a personal problem for you?	
Often	11 (11.0)
Sometimes	20 (20.0)
Occasionally	24 (24.0)
Rarely	37 (37.0)
Never	8 (8.0)
Do you think your shyness can be overcome?	
Yes	72 (85.7)
Uncertain	12 (14.3)
No	0 (0.0)

**Table 2:** Students self-report of shyness affecting their educational experiences.

Item	Yes	No
Speaking up in class	52 (52%)	48 (48%)
Participating in student organizations/sports	33 (33%)	67 (67%)
Asking for letters of recommendations	21 (21%)	79 (79%)
Seeking advice and/or assistance from teachers outside of the classroom	31 (31%)	69 (69%)
Asking questions during class	55 (55%)	45 (45%)
Participating in group discussions/projects	22 (22%)	78 (78%)
Giving a presentation to the class	45 (45%)	55 (55%)
Making friends with classmates	43 (43%)	57 (57%)
Asking for help from other classmates	36 (36%)	64 (64%)
Meeting new people	53 (53%)	47 (47%)
Developing friendships	39 (39%)	61 (61%)
Maintaining relationships	16 (16%)	84 (84%)

**Table 3:** Do any of the following people make you feel shy?

Item	Yes	No
Family members	6	94
Close friends	0	100
Acquaintances	72	28
Strangers	73	27
Authority figures (e.g., professors)	63	37
Classmates	50	50
